# Long-term outcomes of children undergoing video-assisted gastrostomy

**DOI:** 10.1007/s00383-016-4001-3

**Published:** 2016-11-02

**Authors:** Martin Salö, Ana Santimano, Sofia Helmroth, Pernilla Stenström, Einar Ólafur Arnbjornsson

**Affiliations:** Skåne University Hospital and Lund University, Lund, Sweden

**Keywords:** Gastrostomy, Laparoscopy, Children, Outcome, Long term, Gastroraphy

## Abstract

**Purpose:**

The aims of this study were to assess the short- and long-term complication rates after video-assisted gastrostomy (VAG), the effects of age and gender on long-term complications and the effect of duration of gastrostomy tube retention on the need for gastroraphy when the gastrostomy device was removed.

**Methods:**

This was a retrospective study of children undergoing VAG at a single institution. Children who died or moved from the area were excluded. The rates of short- and long-term complications developing at 3–6 months or 2 or more years, respectively, were compared.

**Results:**

A total of 170 children were studied, out of a cohort of 303 children. The median age at surgery was 2 years. The median duration of postoperative long-term follow-up was 5 years (2–9 years). The complications at the respective short and long-term follow-ups were as follows: granulation tissue, leakage, infection and vomiting. There were no differences in the short- versus long-term complication rates for gender and age. Children needing gastroraphy had used a gastrostomy device significantly longer compared with children with spontaneous closure.

**Conclusion:**

Complications after VAG decrease over time. A longer duration of gastrostomy device retention leads to increased need for gastroraphy.

## Introduction

Previous studies have reported on short-term postoperative complications in children undergoing video-assisted gastrostomy (VAG) [1–9]. Problems developing over the long-term remain unknown. This study report will provide information on the long-term outcomes after VAG, which might aid in surgical planning and in the preoperative education of parents. The data should also be useful for estimating the additional burden on medical care following a VAG procedure.

The primary aims of this study were to assess the short and long-term complication rates after VAG, and the effect of age and gender on long-term complications. The secondary aim was to assess the effect of the duration of gastrostomy device retention on the need for gastroraphy when the gastrostomy device was removed.

## Materials and methods

### Setting and pediatric patients

All the enrolled children underwent surgery at a tertiary pediatric surgery center that serves an area with 2 million residents.

### Study design

This was a single-institution, retrospective study. All children <15 years of age who underwent VAG from January 1, 2008 until June 1, 2014 were included. Exclusion criteria were closure of the gastrostomy, death, or relocation out of the study area.

### Operative procedure

VAG entailed the laparoscopy-assisted placement of a Mic-Key^®^ gastrostomy button (provided by Halyard Health, Inc. USA). The VAG operation was performed as described in previously published article [1]. Preoperative antibiotic prophylaxis was given. Through a lower umbilical skin incision, a mini laparotomy was done. A 3–5 mm VersaStep^®^ troacar was safely inserted into the abdomen and pneumoperitoneum was established with CO_2_ insufflation. Using a 3–5 mm 30° laparoscope the stomach was identified. In between the left costal margin and the umbilicus a skin incision was made destined for the gastrostoma. A 5 mm troacar was inserted at this point, through the rectus muscle, into the abdominal cavity under visual control with the laparoscope. Through this port the anterior stomach wall was grasped with an instrument, with clear margins from the pylorus, and exteriorized when the grasper and troacar were pulled back. The stomach was then sutured to the rectus muscle fascia, using a continuous double U stitch, performing a purse string suture around the gastrostoma opening on the stomach wall, and the gastrostomy tube inserted into the cavity of the stomach through a small incision in the stomach wall. The placement of the gastrostomy tube was then controlled gastroscopically at the end of the surgical procedure.

Gastroraphy was performed if spontaneous closure of the stoma did not occur at 1 month or longer after the button was removed.

### Data collection and follow up

The participants were prospectively followed, and postoperative data on complications developed during one of the first 3–6 months were collected.

Information was retrieved from the latest notes, during the latest month, in the prospectively collected medical charts, a median of 5 (2–9) years postoperatively.

The following types of data were collected: age, gender, type of complication, death, relocation from study area, if the gastrostomy button had been removed, and if gastroraphy was required.

The following complications that developed over the short and long-term were recorded: granuloma, infection requiring antibiotic treatment, leakage, vomiting, pain, and dislocation of the button. Complications at long-term follow-up were registered as any during the last 1 month of follow-up.

### Statistical analysis

Clinically significant relevance was considered to be a 10% decrease in the complication rate over time between short and long-term follow-up. The intention was to study independent cases and use each patient as his or her own control. Prior data [1, 2] indicate that the probability of exposure among controls is 0.25. If the true probability of exposure among cases was 0.1, we needed to study 113 patients at short-term follow-up and 113 controls at long-term follow-up to be able to reject the null hypothesis that the exposure rates for cases and controls were equal, with a probability (power) of 0.8. The Type I error probability value for the null hypothesis was 0.05. The Fisher’s exact test was used to evaluate the null hypothesis.

### Ethical considerations

The study was performed according to the Declaration of Helsinki and approved by the Regional Ethical Review Board (registration numbers 2010/49 and 2014/219). The data were coded and de-identified.

## Results

**Fig. 1:**
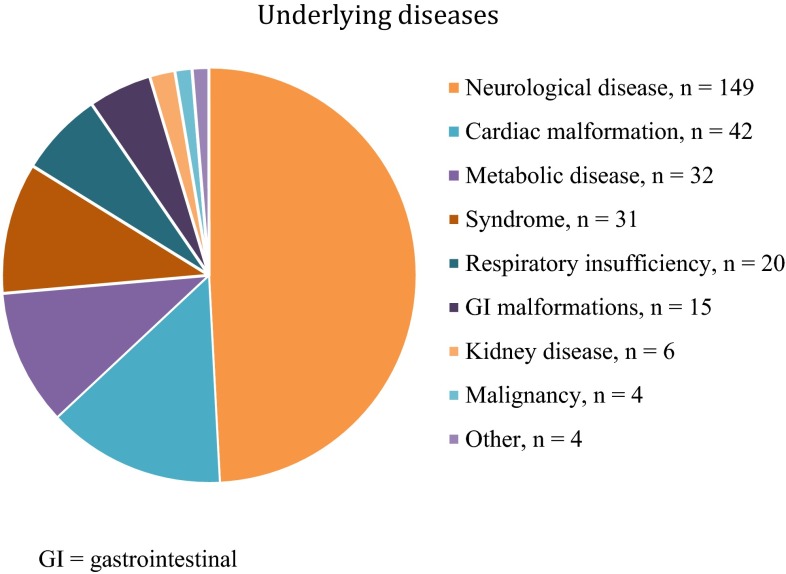
Underlying diagnoses in 303 children undergoing VAG. *GI* gastrointestinal

A total of 303 pediatric patients underwent VAG during the study period and were included in the follow-up. There were 162 boys and 141 girls, with a median age of 1.3 years (1 month to 15 years) at the time of surgery. In children with serious malformations where it was obvious at an early stage that a gastrostomy was needed for a long time or permanent, the operation was performed as early as at the age of 1 month. The majority (*n* = 149, 49%) had an underlying neurological disease (Fig. 1). Many of the children had more than one diagnosis. The most important diagnosis for each child was selected.

**Fig. 2:**
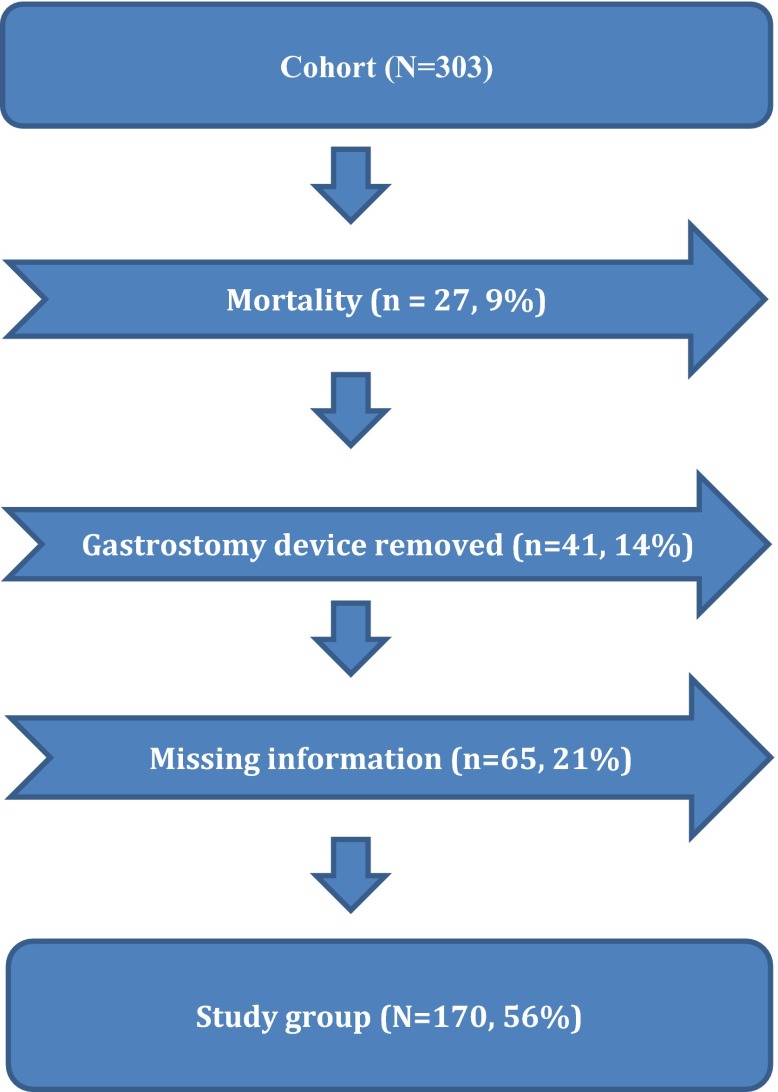
Flow chart describing allocation of study patients for long-term follow-up after gastrostomy

Excluded at the long-term follow-up were the children who had died, no longer had a gastrostomy button, and those whose data were missing (Fig. 2). The deaths were due to the children underlying diseases. There were no deaths due to VAG or aspiration secondary to vomiting. The children, where the data was missing do live in other regions of the country and were not followed-up at our center. Thus, a total of 170 (95 boys and 75 girls) children, were finally evaluated with regard to their short- and long-term complications. The median age at the time of surgery was 2 years (range 1 month to 15 years). The short-term complications were those occurring during a median of 4 (range 3–6) months and the long-term complications were those occurring during a median of 5 (range 2–9) years postoperatively.

A comparison of the short- versus long-term complications revealed significant decreases in the long-term frequencies of minor granulation tissue in most needing local treatment only, minor leakage only, infection, and vomiting (*P* < 0.01). The differences between the rates of tube dislodgement and pain were not significant (Table 1). The tube dislodgement rate was the same at the two episodes of study, during 1 month during 3–6 months postoperatively and during 1 month, 5 (range 2–9) years postoperatively. None of the patients in this study had multiple dislodgements during the two episodes of the study. The percentage and number of children with pain discloses the pain described by the children guardians at two episodes, the first during 1 month, 3–6 months postoperatively and the second during 1 month 5 (range 2–9) years postoperatively.

**Table 1:** Complication rates during short- and long-term follow-up periods after insertion of a gastrostomy button

Complications	At 3–6 months, *N* = 170	At 2–9 years, *N* = 170	*P* value^a^
Granulation tissue	73 (43%)	13 (7%)	<0.01
Leakage	24 (14%)	6 (3%)	<0.01
Infection	26 (15%)	7 (4%)	<0.01
Tube dislodgement	8 (5%)	8 (5%)	1
Vomiting	31 (18%)	4 (2%)	<0.01
Pain	1 (1%)	5 (3%)	0.22
Total	163	43	
No complications	39 (23%)	138 (81%)	<0.01

The listed data are numbers of complications; a single patient can have more than 1 complication

^a^Fisher’s exact test

The difference between the frequencies of complications at long-term follow-up according to gender was not significant (Table 2). Children <2 years of age had a significantly higher frequency of vomiting than the older children (Table 3). No other differences were noted.

**Table 2:** Differences between rates of long-term complications after VAG according to gender

Complications	Boys, *n* = 95	Girls, *n* = 75	*P* value^a^
Granulation tissue	4 (4%)	9 (12%)	0.08
Leakage	4 (4%)	2 (3%)	0.70
Infection	4 (4%)	3 (4%)	1
Tube dislodgement	6 (6%)	2 (3%)	1
Vomiting	2 (2%)	2 (3%)	0.31
Pain	3 (3%)	2 (3%)	1
Total	23	20	
No complications	76 (80%)	62 (83%)	0.81

Numbers are absolute numbers

^a^Fisher’s exact test

**Table 3:** Differences between rates of long-term complications [median follow-up time of 5 years (range 2–9 years)] after VAG according to age at surgery

Complications	<2 years at surgery, *N* = 32	≥2 years at surgery, *N* = 138	*P* value^a^
Granulation tissue	3 (9%)	10 (7%)	0.71
Leakage	0 (0%)	6 (4%)	0.36
Infection	0 (0%)	7 (5%)	0.35
Tube dislodgement	2 (6%)	6 (4%)	1
Vomiting	3 (9%)	1 (1%)	0.02
Pain	1 (3%)	4 (3%)	1
Total	9	34	
No complications	26 (81%)	111 (80%)	1

^a^Fisher’s exact test

**Fig. 3:**
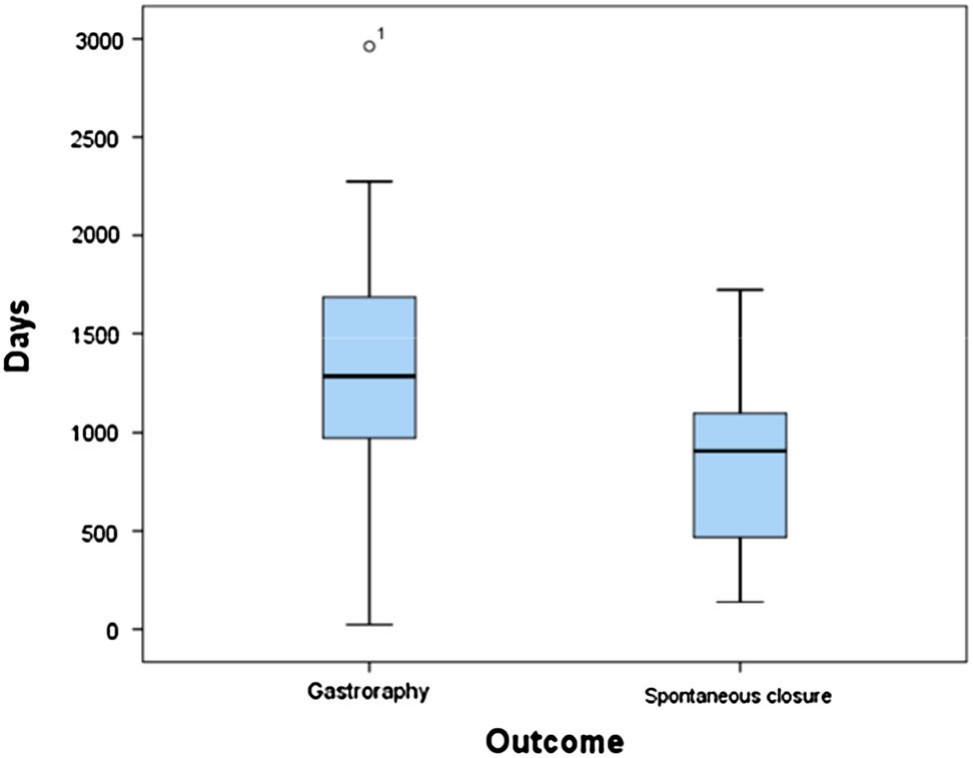
Box-plot showing duration of time after video-assisted gastrostomy (VAG) for removal of the gastrostomy device and spontaneous closure or gastroraphy (*n* = 41, 14% of 303) after a median postoperative observation period of 5 (range 2–9) years. The median duration of retention of the gastrostomy button was 3.5 years (range 1 month to 8.1 years) for children requiring gastroraphy and 2.5 years (range 5 months to 4.7 years) for children with spontaneous closure (*P* = 0.01)

**Fig. 4:**
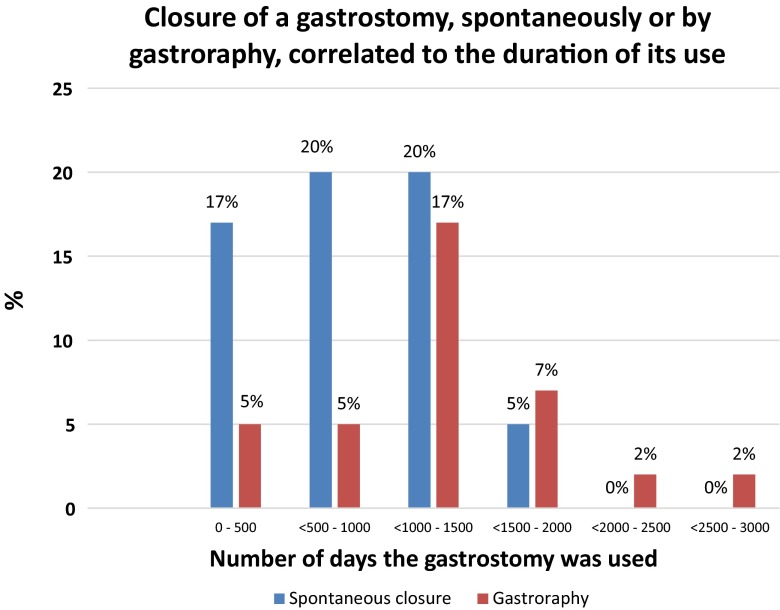
A staple diagram showing the distribution of the number of days using the gastrostomy, divided into two groups based on the removal method, spontaneous closure or gastroraphy (*n* = 41, 14% of 303) after a median postoperative observation period of 5 (range 2–9) years. The median duration of the retention of the gastrostomy button was 3.5 years (range 1 month to 8.1 years) for children requiring gastroraphy and 2.5 years (range 5 months to 4.7 years) for children with spontaneous closure (*P* = 0.01)

Of 41 children who had their gastrostomy button removed during the study period, 25 (61%) developed spontaneous closure, and the rest required gastroraphy (Fig. 2). The median duration of the retention of the gastrostomy button was 3.5 years (range 1 month to 8.1 years) for children requiring gastroraphy and 2.5 years (range 5 months to 4.7 years) for children with spontaneous closure (*P* = 0.01) (Figs. 3, 4).

## Discussion

The frequencies of several postoperative complications of VAG, which developed during a short-term follow-up period in our pediatric study patients, were reduced over a longer time period. The occurrence rates of infection, leakage, granulation tissue, and vomiting were higher during the short-term follow-up than during the long-term follow-up. The frequencies of pain and tube dislodgement did not change over time. Our long-term findings could not be compared with data from previous studies, because no published reports were available.

In general, with the exception of pain and dislodgement of the gastrostomy device, complications due to VAG should be expected to diminish over time. That the frequency of tube dislodgement did not change over time might be accounted for by the care that guardians and patients tend to take with the button, regardless of the duration of time after surgery. This theory is supported by the low frequencies of dislodgement (5%) seen during both the short- and long-term follow ups.

That the frequencies of reported pain did not change significantly over the short- and long-term might be accounted for by the very low frequencies (1 and 3%, respectively) and because the gastrostomy button is a foreign device inserted through the skin and would cause discomfort regardless of the length of time it was in place. Pain is a relative symptom and can be difficult to pinpoint, and communication with a pediatric patient may be especially difficult. Therefore, the treating physician must rely on information from each patient’s guardians.

There were no significant differences between the rates of VAG-related long-term complications for patients stratified by gender. Furthermore, with the exception of more vomiting in children aged <2 years, no difference was found between the rates of long-term complications in children stratified by age, into children younger than 2 years and those 2 years of age and older. In infants, vomiting can be a consequence of other underlying problems or physiologic gastroesophageal reflux, and it is rarely a complication of the gastrostomy device only [10].

Other complications such as the buried bumper syndrome were not seen in our study children. The buried bumper syndrome is a severe complication of the percutaneous endoscopic gastrostomy method, in which the internal fixation device migrates alongside the tract of the stoma outside the stomach [11].

The study found that patients needing gastroraphy had used the gastrostomy button for a longer time than the patients with spontaneous closure of the gastrostomy. This finding is in accordance with previous reports [12]. In addition, the patients needing gastroraphy were older because they had used the gastrostomy button longer, and being older might have negatively affected spontaneous closure. Other factors, including wound healing, medications such as cortisone, and the size of the gastrostomy, probably could also affect the chance of spontaneous closure. Differences in complication rates might have also been similarly affected by underlying diagnoses, medications, and the size of the feeding tube. However, we did not evaluate these variables in this study.

A previous study [12] aimed to test the hypothesis of whether it is possible for the surgeon to decide which stoma has to be closed with a gastroraphy and which to leave for a spontaneous closure, resulted in rejection of the hypothesis of predictability of the gastrostoma closure. The institutional routine at that time was to wait at least 3 months after the removal of the gastrostomy device before suggesting to the child’s guardians an operative closure of the stoma.

The study [12] disclosed that out of a cohort of 321 patients, who had been operated with a video-assisted gastrostomy, 48 children had their gastrostomy button removed. These children were carefully followed and the closure of the gastrostoma was registered. The result showed that in 26 patients the stoma closed within median 3 months (range 1–11 months), whereas in 22 patients a surgical gastroraphy was performed median 4 months (range 2–24 months). There were no differences between the two groups regarding the patients’ diagnoses, the duration of the gastrostoma use or patient’s age at the time of removal of the gastrostomy device. Thus, the authors recommended a routine expectance after the removal of a gastrostomy device for at least 1 month. If no spontaneous closure occurs, then a gastroraphy should be performed. After having tried conservative measurements, the indications for the operative interventions were no other than minor leaks from the gastrostoma.

The results of the long-term follow-up presented here disclose the children problems at the endpoint of the study. During the observation period children without some problem sometimes during the observation period are nonexistent. The scrutinizing of these is out of the scope of this report.

The strengths of this study are the large number of patients and the long follow-up period. Furthermore, all the children had undergone the same standardized VAG procedure, which was performed by the same team of surgeons [1]. All the included children were treated and followed-up at the institution or in institutions sharing the same system of electronic files for patient information. Thus, the reason for the reported decrease in complications cannot be found in lack of information at follow-up.

The study limitations include the subjective reporting and observations and individual interpretation. Information from patients’ charts was prospectively collected but retrospectively compiled. Complication rates might have been impacted, since different treating physicians might have different interpretations and methods of documenting. Notations on the charts were performed without any knowledge that information would be collected for a retrospective study, and therefore, might have been not focused on the gastrostomy device. In addition, complications such as intermittent infections and leakage over a long time-period might not have been noted in the charts, and therefore, not have been included in the evaluations.

The results of the study will mainly serve as information for physicians and other healthcare workers that can be referred to when informing children and their guardians on the frequency of complications and types of outcomes after VAG surgery. The results might also be taken into account when deciding whether or not gastroraphy should be performed. The data should also be useful for estimating the additional burden on a hospital and outpatient care following a VAG procedure. Future prospective studies that provide more accurate results and are less influenced by bias are warranted.

## Conclusion

Complications after VAG in children decrease over time. A longer duration of gastrostomy device retention leads to a decreased probability of spontaneous closure if the device is removed. These findings provide important information for parents preoperatively and for clinical follow-up postoperatively.
